# Factors influencing acceptance, adoption and adherence to sentinel node biopsy recommendations in the Australian Melanoma Management Guidelines: a qualitative study using an implementation science framework

**DOI:** 10.1186/s43058-022-00351-w

**Published:** 2022-10-01

**Authors:** Andrea L. Smith, Caroline G. Watts, Michael Henderson, Georgina V. Long, Frances Rapport, Robyn P. M. Saw, Richard A. Scolyer, Andrew J. Spillane, John F. Thompson, Anne E. Cust

**Affiliations:** 1 The Daffodil Centre, The University of Sydney, a joint venture with Cancer Council NSW, Sydney, NSW Australia; 2 Australian Institute of Health Innovation, Macquarie University, Sydney, NSW Australia; 3 Surveillance, Epidemiology and Research Program, Kirby Institute, University of New South Wales, Sydney, NSW Australia; 4 Peter MacCallum Cancer Centre, Melbourne, VIC Australia; 5 Melanoma Institute Australia, The University of Sydney, Sydney, NSW Australia; 6 Faculty of Medicine and Health, The University of Sydney, Sydney, NSW Australia; 7 Department of Medical Oncology, Royal North Shore Hospital, Sydney, NSW Australia; 8 Mater Hospital, Sydney, NSW Australia; 9 Charles Perkins Centre, The University of Sydney, Sydney, NSW Australia; 10 Royal Prince Alfred Hospital, Sydney, NSW Australia; 11 NSW Health Pathology, Sydney, NSW Australia; 12 Department of Breast and Melanoma Surgery, Royal North Shore Hospital, Sydney, NSW Australia

**Keywords:** Sentinel lymph node biopsy, Clinical practice guidelines, Melanoma, Implementation science framework, Professional groups

## Abstract

**Background:**

Sentinel node biopsy (SN biopsy) is a surgical procedure used to accurately stage patients with primary melanoma at high risk of recurrence. Although Australian Melanoma Management Guidelines recommend SN biopsy be considered in patients with melanomas > 1 mm thick, SN biopsy rates in Australia are reportedly low. Our objective was to identify factors impacting the acceptance, adoption and adherence to the Australian SN biopsy guideline recommendations.

**Methods:**

Opinions of Australian key informants including clinicians, representatives from melanoma education and training providers, professional associations and colleges, and melanoma advocacy organisations were collected through semi-structured interviews (*n* = 29) and from publicly released statements (*n* = 14 news articles). Data analysis involved inductive and deductive thematic analysis using Flottorp’s determinants framework.

**Results:**

A complex interplay of contemporary and historical factors was identified as influencing acceptance, adoption and adherence to the SN biopsy guideline recommendations at the individual, guideline, patient, organisational and social levels. Expert and peer opinion leaders have played an important role in facilitating or inhibiting adoption of guideline recommendations, as have financial incentives driven by healthcare-funding policies and non-financial incentives including professional identity and standing. Of critical importance have been the social and knowledge boundaries that exist between different professional groups to whom the guidelines apply (surgeons, dermatologists and primary care practitioners) with adherence to the guideline recommendations having the potential to shift work across professional boundaries, altering a clinician’s workflow and revenue. More recently, the emergence of effective immunotherapies and targeted therapies for patients at high risk of recurrence, the emergence of new opinion leaders on the topic (in medical oncology), and patient demands for accurate staging are playing crucial roles in overcoming the resistance to change created by these social and knowledge boundaries.

**Conclusions:**

Acceptance and adherence to SN biopsy guideline recommendations in Australia over the past 20 years has involved a process of renegotiation and reframing of the evidence for SN biopsy in melanoma by clinicians from different professional groups and networks. This process has helped to refine the evidence for SN biopsy and our understanding of appropriate adoption. New effective systemic therapies have changed the balance towards accepting guideline recommendations.

**Supplementary Information:**

The online version contains supplementary material available at 10.1186/s43058-022-00351-w.

Contributions to the literature

- Evidence plays a critical role in the adoption of innovations. However, interpretation of the evidence and acceptance of an innovation is as much a social as a scientific process
- We found that social and knowledge boundaries between dermatologists, surgeons and primary care physicians played a critical role in shaping how evidence for SN biopsy was interpreted, generating intense debate about its role.
- The emergence of new evidence (effective adjuvant systemic therapies) and new opinion leaders from medical oncology have played an important role in disrupting the social and knowledge boundaries between professional groups and communities of practice in melanoma management.

## Background

Melanoma is the third most common cancer diagnosis in Australia [1]. Survival is influenced by the stage of the melanoma at diagnosis. Wide local excision is the standard treatment for early cutaneous melanomas, and for most patients is curative. Some patients, however, will subsequently relapse with regional or more distant recurrence. After definitive wide excision of a primary melanoma, the first site of recurrence is most likely to be in the regional lymph nodes [2].

Identification of patients who are at increased risk of recurrence requires accurate cancer staging and relies on the international AJCC (American Joint Committee on Cancer) staging system for cutaneous melanoma. Melanoma staging is based on tumour thickness (T), subcategorised by the presence or absence of tumour ulceration, lymph node spread (N) and distant metastasis (M) [3–5].

Guidelines for sentinel node (SN) biopsy were first introduced in Australia in 1999 and have been updated several times since then (Table 1). In 2017, the guidelines were made available online. The Australian Melanoma Management Guidelines currently recommend that SN biopsy be considered for all patients with melanoma > 1.0 mm in thickness or 0.8–1.0 mm with high-risk pathological features (e.g. ulceration or lymphovascular invasion) [6].

**Table 1:** Australian Clinical Practice Guidelines for the diagnosis and management of melanoma

1999: Lymphatic mapping and sentinel node biopsy should be considered for all melanomas > 1.0 mm thick provided they can be done in the context of a controlled clinical trial and by surgeons trained in these procedures
2008: Patients with a melanoma > 1.0 mm in thickness should be given the opportunity to discuss sentinel lymph node biopsy to provide staging and prognostic information
2018: Sentinel lymph node biopsy should be considered for all patients with melanoma > 1.0 mm in thickness and for patients with melanoma > 0.8 mm with other high risk pathological features to provide optimal staging and prognostic information and to maximise management options for patients who are node positive

SN biopsy is currently the most sensitive method of detecting clinically occult nodal disease at the time of diagnosis of the primary melanoma and is recommended for staging of patients with higher-risk primary melanomas [6]. Patients initially staged clinically as Stage IB, IIA,B,C, that is, they have no clinical evidence of lymph node involvement, will be upstaged to Stage III if a SN node biopsy detects microscopic evidence of metastatic melanoma. The risk of lymph node spread varies from at least 5% for patients whose melanoma is 0.8–1.0 mm in thickness with ulceration to over 25% for patients with ulcerated thick (> 4 mm) melanomas, although other factors such as patient age also influence SN metastasis risk. Several nomograms exist that provide a more individualised assessment of the risk of having a tumour-positive SN [7, 8].

Adjuvant systemic therapies including immunotherapy and targeted therapies have been shown to be effective at reducing rates of recurrence in melanoma patients whose tumour has spread to locoregional lymph nodes (high-risk patients such as those with Stage IIB, IIC and III melanoma) [9–11]. In Australia, adjuvant immunotherapy and targeted therapy are both available for patients with resected Stage IIIB-IIID melanoma at no cost to the patient through the national Pharmaceutical Benefits Scheme (PBS). Accurate staging allows the identification of patients most likely to benefit from adjuvant systemic therapy and more intensive follow-up.

Factors influencing use of SN biopsy are poorly documented in the Australian context. The limited data that exist suggest that rates of SN biopsy for melanoma in Australia may be lower than the guidelines suggest they should be. The 2006–2007 New South Wales Melanoma Patterns of Care Study reported that SN biopsy was performed in 45% of patients diagnosed with a melanoma > 0.75 mm thick [12, 13]. Given that appropriate treatment and management is increasingly dependent on accurate staging, it is important to understand why rates might be low. Our previous research in Australia has shown that although there is ‘in principle’ support of the role of SN biopsy in accurately staging patients with melanoma, some dermatologists express disagreement with and distrust of the guideline recommendations for SN biopsy [14], and primary care physicians commonly have low levels of understanding of the recommendations [15]. These knowledge gaps and distrust of the recommendations are potentially concerning as primary care physicians and dermatologists are typically the first point of contact for people diagnosed with melanoma [16].

Clinical practice guidelines synthesise and summarise complex research evidence into easily understandable recommendations. However, it is recognised that the distillation and summary of evidence into clinical practice guidelines, although a necessary step, is not in and of itself necessarily sufficient for the translation of research evidence into routine clinical practice [17–19]. In addition to the strength and quality of the evidence and the recommendations, moving evidence into practice within a particular setting or within a particular group of individuals is impacted by other factors. These include the influence of key opinion leaders including peer or expert opinion leaders (e.g. academics or senior clinicians) [20].

The purpose of this study was to better understand factors influencing adherence to the SN biopsy recommendations. Implementation science offers theory-informed means of systematically interrogating an evidence-to-practice gap. Our aim was to identify, from the perspective of key informants, factors that they believed were impacting the current and future use of SN biopsy in the clinical management of invasive melanoma in Australia. We anticipated that these factors would be diverse, operating at the level of the intervention (i.e. the clinical practice guidelines), the individual (i.e. the clinician) and at a broader more organisational or societal level. We therefore used the Flottorp determinants of practice implementation science framework as a major overarching framework to inform our data collection, analysis and reporting [21]. Flottorp’s framework is a meta-theoretical implementation science framework that draws on twelve implementation frameworks, including the Diffusion of Innovations in Service Organizations [22], the Consolidated Framework for Implementation Science [23] and the Theoretical Domains Framework [24]. In the discussion of our findings, we considered in more detail the literature on organisation and business management [25], innovation in service delivery and organisation [22], and de-implementation [26, 27]. We have drawn on practice-based theories which propose that knowledge is localised, embedded and invested in collective practice and that boundaries are inherent sociocultural differences between distinct collective practices underpinned by shared language, meanings and ways of doing things [28, 29]. In particular, we have drawn on Wenger’s communities of practice approach [30], Ferlie’s uni-professional communities of practice [25] and Gabbay and le May’s multi-professional communities of practice [31].

## Methods

### Study design

This qualitative interview and document-based study was approved by the University of Sydney Human Research Ethics Committee (Protocol Number: 2018/713).

### Participants and recruitment

The project team (comprising dermatologists, surgeons, medical oncologists, epidemiologists, health services researchers and melanoma researchers) developed a list of key informants with expertise in melanoma in Australia based on the team’s knowledge of the subject area and recognition of national figures working across sites and settings including support organisations and consumers. Consumers included consumers’ representatives (i.e. people who have had a melanoma diagnosis) and executives within consumer advocacy organisations. Potential participants were purposively selected to ensure wide-ranging views from melanoma consumer organisations and from clinical specialties including surgery, dermatology, primary practice, medical oncology and pathology. Purposively sampling meant that a wide range of participants was initially invited to participate, with subsequent interviews focusing on those stakeholders with clinical backgrounds found to be most relevant to the research question, i.e. dermatologists, surgeons and primary care physicians. In addition to their clinical or consumer knowledge and expertise, almost all participants held senior or executive positions within melanoma education, training and advocacy organisations, professional associations and colleges. To ensure key informants were not limited to those already known to the research team, we searched and identified individuals who had commented publicly on SN biopsy in Australia using Factiva, an online database of news and business information [32]. Personalised emails were sent to each potential participant explaining why they had been selected, the relevance of the study and the importance of capturing their perspectives. Of the 38 key informants invited to take part in the study, 29 (76%) agreed to be interviewed. Several of those who were invited did not respond to the email contact; of those who responded and declined, the most commonly stated reason for declining was lack of time. Participants were also asked to nominate additional individuals they believed should be included in the study.

### Data collection

A semi-structured interview guide was developed (Supplementary file) with input from the research team based on the study objectives. The interview guide focused on understanding the specific factors that key informants believed were affecting decision-making and attitudes towards SN biopsy adoption amongst clinicians involved in the management of invasive melanoma. Questions examined four areas: (1) past, current and future use of SN biopsy in melanoma management; (2) the diverse nature of opinions in relation to SN biopsy for melanoma management; (3) the reasons for these differing opinions; and (4) the barriers and facilitators to implementation of SN biopsy guideline recommendations in melanoma management.

To maximise the breadth of informant opinion and to complement the interview data, news stories identified via Factiva that related to use of SN biopsy in Australia were also included. Searches were undertaken to identify public statements relating to the use of SN biopsy in the management of melanoma in Australia. The search terms ‘sentinel lymph node biopsy’, ‘sentinel node biopsy’, ‘SLN biopsy’ and ‘SN biopsy’ were combined with ‘melanoma’. Searches were limited to 1990–2021 (the period in which SN biopsy has been available) and to the Australian news media.

### Data analysis

Analysis of the documents and interview transcripts was informed by a social constructivist approach and drew on analytical techniques used in reflexive thematic analysis [33, 34] and grounded theory, particularly constant comparison [35]. Our social constructivist approach allowed us to explore the subjective and complex experiences of participants. In the context of the study, social constructions included participants’ knowledge and beliefs about SN biopsy and the SN biopsy recommendations. The constant comparison technique was used to identify commonalities and differences across interviews and news story data. Reflexive thematic analysis ensured that we went beyond describing what we found, integrating and analysing our findings inductively.

Interview data and the news story data from the Factiva searches were first analysed inductively to develop themes relating to key informants’ perspectives on use of SN biopsy. The developing themes were assessed to ensure they were supported by data from the interviews and the news stories, which provided a form of triangulation. Data were then analysed deductively using the Flottorp determinants of practice framework [21], which provided a systematic means of understanding current and future determinants of practice in relation to SN biopsy. Analysis involved two researchers (ALS and CW) reading the de-identified transcripts. Coding and theme development were performed by ALS. NVivo 12 software was used to support coding, data management and data retrieval. The data from the 29 interviews and the 14 news stories were compared to identify commonalities, differences and patterns. The themes were then discussed and refined with members of the research team until agreement was reached on the themes. Analysis drew on a range of theoretical resources and sensitising concepts that were identified as relevant and useful in moving the analysis from description to interpretation, including implementation science [17, 21, 26, 27], organisational change in professionalised organisations [25], and sociology [22, 36]. Findings were reported according to the SRQR guidelines [37].

### Reflexivity

Throughout the study, a research journal was kept logging decisions, thoughts and reflections relating to all stages of the project. In particular, the journal was used to (1) log how existing relationships between members of the research team and study participants might be impacting on data collection and analysis and (2) document how these relationships, the research team’s backgrounds and pre-existing knowledge might be contributing to the construction of particular narratives during data collection and analysis and the reporting of our findings.

## Results

Twenty-nine key informants (25 with a clinical background; 4 with a consumer perspective) were interviewed between May 2019 and October 2020, continuing to recruit until no new information was generated (i.e., thematic saturation). The characteristics of the key informants are shown in Table 2.

**Table 2:** Characteristics of participants (*n* = 29)^a^

Characteristic	Number (%)
**Gender**	
Male	26 (90)
Female	3 (10)
**Clinical background**^**b**^	
Dermatology	12 (41)
Primary care	5 (17)
Surgery	5 (17)
Medical oncology	3 (10)
Pathology	1 (3)
Non-clinical^c^	4 (14)
**Professional role**^**d**^	
Practising or retired clinician	25 (86)
Senior leadership position within an organisation providing post-graduate skin cancer education (academic or private)	5 (17)
Senior leadership position within a consumer/research organisation	4 (14)
Representative of a professional organisation	4 (14)
**State**^**e**^	
New South Wales	12 (41)
Victoria	10 (34)
Queensland	5 (17)
Western Australia	1 (3)
Australian Capital Territory	1 (3)

^a^ Participants were purposively sampled based on their experience in relation to melanoma in Australia, for example holding senior positions within professional colleges and associations, melanoma units or skin cancer organisations, involvement in large-scale clinical trials or clinical guideline development, involvement in skin cancer training and education

^b^ One clinician had worked both as a specialist and as a primary care physician

^c^ Non-clinical backgrounds included consumer representatives and executives from consumer advocacy organisations

^d^ Several participants had more than one role

^e^ Four of the participants had (or had previously held) senior or executive roles representing national organisations

The Factiva searches identified 89 results, of which 14 were relevant to the objectives of this study. These media reports presented the views of a small number of experts (primarily clinicians (*n* = 8), but also researchers (*n* = 2) and patient advocates (*n* = 2)) and all related to the use of SN biopsy in Australia. All of the views reported in the Factiva results were corroborated by the key informant interviews. One key informant who had not previously been known to the research team was identified through the Factiva searches. This person was invited to participate but declined to do so.

### Factors influencing acceptance, adoption and adherence to SN biopsy guideline recommendations

**Fig. 1:**
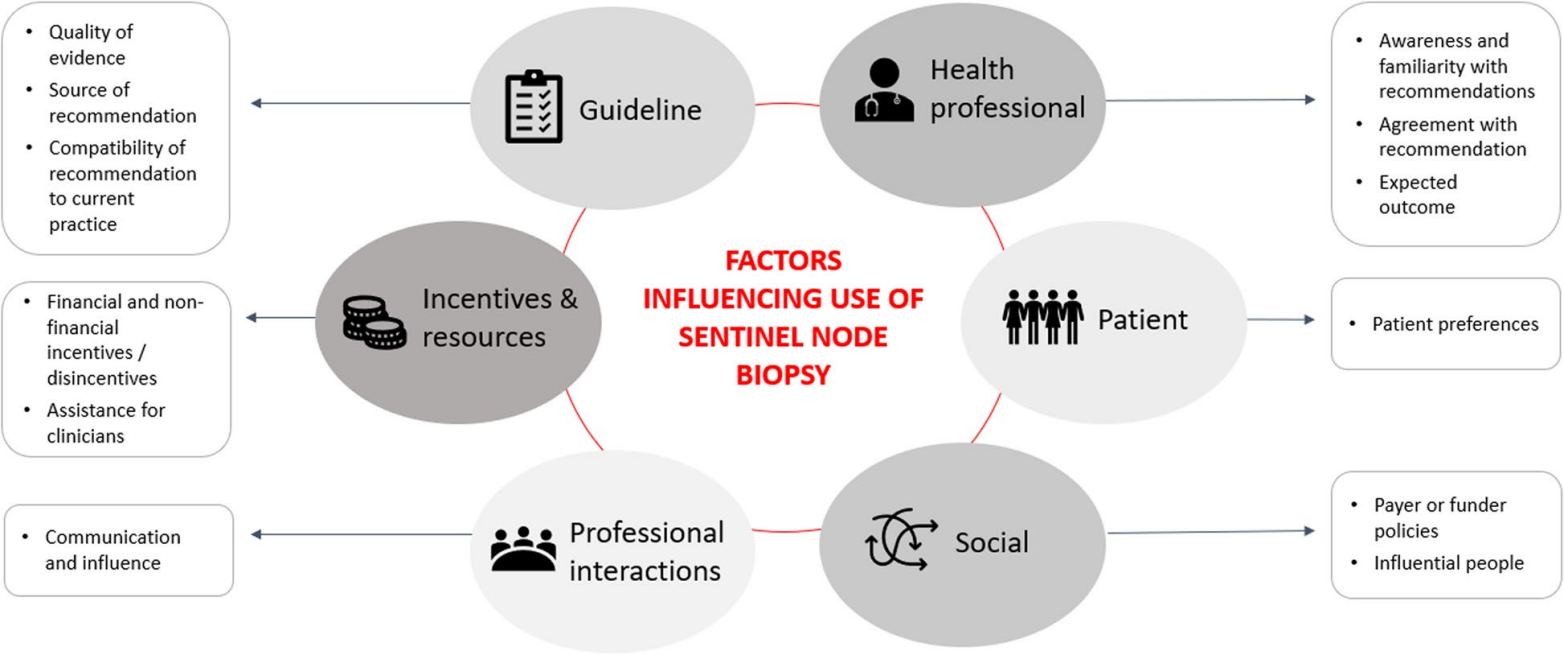
Twelve determinants across six of the Flottorp domains [21] were identified as influencing acceptance and adoption of the sentinel node biopsy guideline recommendations in management of patients with primary melanoma

Twelve determinants across six of the seven Flottorp domains were identified as potentially influencing acceptance, adoption and adherence to the SN biopsy guideline recommendations in Australia (Fig. 1; Supplementary file Tables S1, S2, S3, S4, S5 and S6). The domains included: guideline factors; health professional factors; patient factors; professional interaction factors; incentives and resource factors; and social factors. Overall, the key influence on acceptance, adoption and adherence to guideline recommendations was the social and knowledge boundaries that exist between professional groups. From the analysis, we identified a number of strategies to support acceptance, adoption and adherence to the SN biopsy guideline recommendations (Table 3).

**Table 3:** Strategies that could support clinician use of SN biopsy guideline recommendations

1.The key informants emphasised that drawing on multidisciplinary expertise not only helped clinicians to keep abreast of the latest developments in melanoma management but that it helped to overcome suspicion of the motivations of other specialties
2.Knowledge dissemination strategies need to consider the important role of professional influence on determining clinicians’ practice, as well as the complex ways in which evidence and clinical experience interact to influence practice. Knowledge dissemination approaches that engage individual healthcare professionals and local groups and leverage the influence of expert and peer opinion leaders are more likely to facilitate attitude change rather than top-down, policy-driven changes
3.State-based data on rates of sentinel node biopsy (SN biopsy) in Australia might assist in service planning for treatment of melanomas
4.The role of SN biopsy requires careful messaging, in particular: • SN biopsy is a staging procedure, and it is accurate staging that allows the identification of patients who might benefit from systemic therapy and more intensive follow-up • Avoiding language that frames SN biopsy as being the ‘gatekeeper’ to systemic therapies, e.g. clinicians often talk about one of the advantages of SN biopsy being that it will ‘allow’ patients access to systemic therapies. Rather, it is a staging procedure, and it is accurate staging that determines future management, including treatment with systemic therapies

Some of the key informants believed that knowledge of and familiarity with the SN biopsy recommendations could be improved particularly amongst primary care physicians (Health Professional Factors – Awareness and Familiarity with Recommendations), and that adherence to the recommendations may be being impacted by the accessibility of the guidelines. A suggested solution included a simple-to-understand summary, decision-making tool or algorithm (Incentives and Resources – Assistance for Clinicians). However, a bigger issue was the level of agreement with the guidelines (Health Professional Factors – Agreement with Recommendation). This was in part due to differing interpretations of the clinical trial evidence, which was often dependent on the professional group to which they belonged.

I don’t like the phrase extremism, what you have is just, I think, two groups of people who have looked at the same literature and reached different conclusions. Then you have a large group of people in the middle who are happy to be guided by the experts. ID3 Dermatologist

Almost all key informants talked about how the at times heated and polarised discussion relating to key clinical trial data (Guideline Factors – Quality of Evidence) had impacted on attitudes and beliefs about SN biopsy, and ultimately on uptake of SN biopsy. Key informants talked extensively about the evolving role of SN biopsy and surgery in melanoma management and the importance of debate and research in shaping past, current and future use of SN biopsy, and in challenging evidence, data, norms, beliefs and practices (Social factors – Influential People). It was acknowledged that while this debate has at times seemed intense and potentially detrimental to improved patient outcomes, ultimately it has been a driving force in refining evidence and knowledge. Several key informants emphasised the important role opponents of SN biopsy have played in ensuring that claims made in relation to SN biopsy’s prognostic value and survival benefit were rigorously tested (Guideline Factors – Quality of Evidence).

Many of the key informants commented that the ambivalence amongst some clinicians, especially dermatologists, towards SN biopsy may have stemmed from a distrust of the scientific evidence around the role of elective lymph node dissection in reducing the risk of recurrence in melanoma. (Guideline Factors – Quality of the Evidence) This distrust was driven in part by what some perceived to have been the over-promotion of elective lymph node dissection, and then SN biopsy, and that many believed some surgeons’ enthusiasm for these procedures exceeded the evidence available potentially evoking a negative response and pushback from other specialties, especially dermatology (Social factors – Influential People).

I fundamentally distrusted the literature and the evidence on the elective lymph node dissection for many of the reasons. It's a procedure that's promoted strongly by the proceduralists who do it and the enthusiasm for the procedure exceeded the evidence. A lot of the literature was biased and full of spin that suggested a benefit to the surgery that wasn't supported by the evidence. ID5 Dermatologist

Several key informants indicated that up until very recently many clinicians, in particular dermatologists and primary care physicians, did not believe that a SN biopsy would result in better patient outcomes and this had had a significant impact on attitudes to the SN biopsy guideline recommendations and therefore clinical practice (Health Professional Factors: Expected Outcomes). Consequently, the landmark MLST-II [38] and DeCog-SLT [39] trials were widely cited by key informants as an example of the importance of evidence generated from rigorous clinical trials, and how this evidence can quickly drive change, in this case, the rapid de-implementation of completion lymph node dissection in patients with a positive SN biopsy and the clarification of whether SN biopsy’s primary role is prognostic or therapeutic (Guideline Factors – Quality of the Evidence).

While it was acknowledged that most clinicians accepted that a SN biopsy could provide additional prognostic information, the key informants reported that the lack of effective treatment options for those found to be SN positive meant many clinicians did not see the value of SN biopsy (Health Professional Factors: Expected Outcomes). Many clinicians did not consider that adherence to the recommended guidelines would lead to better outcomes for their patients. Importantly, most of the key informants reported that the belief that SN biopsy did not contribute to better patient outcomes had shifted with the PBS listing of systemic therapies for high-risk Stage III patients (Guideline Factors – Quality of the Evidence).

A lot of the people in the primary care sector and a lot of people doing a lot of skin work felt that actually [the prognostic information] didn't justify [doing a SN biopsy]. Because of no [improvement in] survival outcomes, it wasn't clearly demonstrated that there was good survival outcome, that [it was] something that should be routinely offered to patients. ID11 Primary care physician

A small number of key informants also commented that some clinicians were wary of the Australian guidelines owing to what some perceived to be conflicts of interest amongst those involved in the guideline development process (Guideline Factors – Source of Recommendation).

I think just the perception from outside of [the guidelines being chaired by a surgeon] is probably not favourable. So I think probably it would have been ideal to have declared that as a conflict and probably stepped aside from any discussions around sentinel node biopsy. ID6 Dermatologist

Other key informants commented that the guideline recommendation impacts on current work practices, in particular the practice point that states that clinicians should refer all eligible patients to a surgeon for discussion of SN biopsy (Guideline Factors – Compatibility of Recommendation to Current Practice). It was also noted that the overlapping roles of surgeons, dermatologists and primary care physicians meant that there was a potential financial disincentive to refer patients to other clinical specialties for ongoing management such as a wide local excision owing to the risk of losing longer-term management of that patient (Incentives and Resource Factors – Financial Incentives and Disincentives).

The practice point [referral to surgeon] gets under the skin of dermatologists. [Dermatologists are concerned as] it says we have to send every patient with melanoma this thick to a surgeon or we may be negligent … I think it is the view amongst some that they’re being dealt out of melanoma management. So dermatologists do feel threatened. ID15 Dermatologist

There are vested interests. It is possible that [dermatologists and primary care physicians] don't wish to refer patients to the surgeon ... You have to do the wide excision at the same time. So, in other words, they’re losing business, losing patients if they refer them. ID22 Surgeon

The Australian healthcare system’s ‘fee for service’ model means that clinicians get paid more for a wide local excision than for a complex clinical consultation that does not involve a procedure (Social Factors – Payer or Funder Policies), and this was identified as a driver of behaviour. However, financial interests were considered only a small part in explaining clinician’s attitudes, beliefs and therefore practice in relation to SN biopsy. Instead, key informants reported that factors such as professional standing, professional identity, personal recognition and academic reputation were critically important (Incentives and Resource Factors – Non-financial Incentives and Disincentives).

A lot of this gets caught up in ego and position and you can’t change your mind, can’t be seen to be changing your mind … some of it is based on insufficient expertise in a particular space, some of it’s based around long-held views, the way people were trained. ID11, Primary care physician

Key informants commented that positions and practices can become entrenched leading to clinical inertia. Many people had publicly adopted a certain stance in relation to SN biopsy, had invested time and resources in developing particular clinical skills, achieved a certain status and derived satisfaction from the procedures that they performed (i.e. wide local excisions and/or SN biopsy) and believed that what they were doing was in the best interests of the patient (Health Professional Factors: Expected Outcomes). Consequently, different normative beliefs existed about SN biopsy, often based on the beliefs and practices of senior clinicians or key opinion leaders within clinicians’ communities of practice and professional networks (Professional Interactions – Communication and Influence). Sometimes, these normative beliefs were out of step with current recommendations and the latest evidence for effective melanoma management.

A lot of what we do in practice reflects what our bosses, when we were trainees, always did, or did routinely, or considered was standard. ID1, Dermatologist

You could ask why doctors, including myself, are so bad at changing … a fair amount of that is compliance. The majority of people would be reluctant, I think, to engage in the friction that results from saying they're actually not going to do what the most eminent person in the land says you should be doing. ID3, Dermatologist

Several of the key informants commented that the growing complexity of melanoma management means that optimal care increasingly demands multidisciplinary team consideration, with support and guidance from pathologists, dermatologists, surgeons, radiation oncologists and medical oncologists. It was noted that multidisciplinary teams, and medical oncologists in particular, were playing a key role in helping to improve knowledge and shift beliefs about the utility of SN biopsy in guiding melanoma management (Health Professional Factors: Awareness and Familiarity with Recommendations; Professional Interactions – Communication and Influence).

I would say it’s education [that has improved attitudes towards and use of SN biopsy]. We, the medical oncologists, have started to push a little bit on that actually, because the reality is because we got access to medications to use. ID28 Medical oncologist

It was also suggested that clinicians who were not connected to multidisciplinary teams were more likely to hold out-of-date beliefs about the current role of SN biopsy in melanoma management.

Key informants acknowledged that some clinicians believed that the guidelines may be too restrictive and some patients with melanoma who were at high risk of metastasis and disease recurrence either fell outside the current guidelines for having a SN biopsy (e.g. some younger patients with melanomas < 0.8 mm) or did not meet the requirements for adjuvant systemic therapy (e.g. some patients with melanomas > 4.0 mm but who had no palpable or occult nodal involvement). It was acknowledged that evidence is still accumulating regarding criteria for a SN biopsy and that the importance of correctly identifying patients for SN biopsy had increased owing to the availability of effective treatments and clinical trials for patients with stage II and III disease. The speed at which evidence was accumulating and knowledge increasing meant that some of the clinicians felt that best practice means practising ahead of the current guidelines and consulting with trusted colleagues.

The other thing is, is that the indicators are constantly changing, you know, who gets [SN biopsy] and who doesn’t. But what I tend to do is that if I’m not sure, I’ll actually run the cases by the melanoma unit or a couple of my colleagues that I work very closely with that are part of the melanoma unit, just to find out what’s actually current, because obviously with the different trials and studies, then the inclusion criteria are modified on a regular basis, and sometimes they’ll be party to information that hasn’t actually hit the mainstream journals and things yet, so I’ll try and keep [us] even more current and up to date than most other people as well. ID23 Surgeon

Some key informants emphasised the importance of considering patient preferences and acknowledging that these may differ from those of the clinician, especially if the clinician holds strong views on the role of SN biopsy in melanoma management (Patient Factors: Patient Preferences). There was an increasing sense that patients diagnosed with melanoma had a right to be staged as accurately as possible.

If you don't know what your stage is then you're potentially not able to access immunotherapy in the adjuvant setting, for example, or other treatment options that might benefit or reduce the risk of the melanoma. They're life or death issues so patients need to be informed and have the options available to them so they can make decisions about their own health. ID13 Melanoma patient advocacy organisation

Many of the key informants therefore stressed that patients needed to be supported to make informed decisions about the utility of SN biopsy and to understand how detection of micro-metastatic disease could impact on disease management, including treatment options that might be offered if micro-metastases were detected in their lymph nodes. Key informants indicated that for some clinicians, this would mean becoming more patient-focused and engaging in shared decision-making rather than making management judgements on behalf of the patient (Patient Factors: Patient Preferences).

There are clinicians, old school, I would say, mostly, who believe that the clinician is always right, my judgement [about SN biopsy] is the best judgement, you don’t need to hear anything else. ID21 Melanoma patient advocacy organisation

[Our role as clinicians is to] make the patient understand why you might be doing [a SN biopsy] - searching for micro-metastatic disease, [that it’s] a staging tool that might upgrade their staging and then - get them closer to treatments - but also triage who we want to monitor more closely. ID19 Dermatologist

## Discussion

This study advances the theoretical debate on adoption of innovations in healthcare [22, 40] by reflecting on the role that professional networks, communities of practice and expert opinion leaders play in ensuring that an innovation is appropriately adopted in melanoma management. We found that the social and knowledge boundaries between dermatologists, surgeons and primary care physicians played a critical role in shaping how evidence for adoption of SN biopsy was interpreted, generating debate about the role of SN biopsy in melanoma management. The different professional networks and communities of practice that exist within the field of melanoma management played a role not just in determining the meaning attached to SN biopsy but also in influencing the sharing and diffusion of knowledge between communities of practice. Consequently, clinicians’ practice was determined as much by what they knew as by the norms that existed within their community of practice. However, seemingly entrenched opinions and behaviours can shift suddenly when evidence from another field, in this instance medical oncology, and new opinion leaders emerge helping to reframe the meaning that clinicians attach to an innovation and the perceived value of that innovation to them and to their patients.

Evidence, even strong evidence, can be perceived to be contestable, necessitating debate and negotiation within and between professional groups such as dermatologists, surgeons and primary care physicians. Consequently, the meaning attached to an innovation such as SN biopsy is generally not fixed. For any potential innovation, the evidence is rarely clear cut. It is known that innovations, even if backed by strong evidence, are not necessarily accepted willingly [36, 41]. The evidence is also often interpreted differently by different stakeholders. Furthermore, these interpretations can change over time. This is in many ways not surprising as the production and interpretation of evidence is a social as well as a scientific process [22, 41].

One of the most powerful forces impacting on the meaning attached to SN biopsy and perceptions of the role of SN biopsy was the existence of professional networks and communities of practice. A community of practice is a group whose shared knowledge, practice and identity creates boundaries that differentiate it from other communities of practice [29, 30]. The current study identified several distinct (but sometimes overlapping) communities of practice involved in managing patients with melanoma in Australia, including dermatologists, surgeons, primary care physicians, multidisciplinary teams (i.e. groups of clinicians from several different disciplines who meet regularly to discuss the care of patients with a particular focus, such as melanoma) and research-focused clinicians. The importance, and limitations, of professional and healthcare communities of practice in promoting and blocking knowledge sharing has been reported in several key studies [25, 28, 29]. In particular, Ferlie et al. have reported that healthcare communities of practice tend to consist of a limited number of different types of healthcare professional, that they seal themselves off from neighbouring communities of practice and that they develop internal learnings while simultaneously blocking externally oriented sources of change and learning [25]. It is also recognised that uptake of new procedures can be rapid amongst homogenous specialty groups with strong opinion leaders in them, with uptake often occurring ahead of the research validating these innovations [42, 43].

This study highlighted the existence of social and knowledge boundaries between the different professional groups to whom the SN biopsy guideline recommendations applied (surgeons, dermatologists and primary care physicians). These boundaries prevented or slowed the spread of knowledge between the different communities of practice. Membership of multiple communities of practice has been reported by others as being crucial for bridging boundaries between communities [44] and might explain the finding in this study of greater acceptance of SN biopsy by dermatologists and surgeons associated with multidisciplinary teams or research-focused cancer centres.

Our study indicates that a community of practice’s normative beliefs can, however, shift suddenly and even unexpectedly when evidence from another field emerges. The introduction of effective adjuvant systemic therapy for the management of melanoma acted as a disruptor, rapidly overturning past hesitancies about the use of SN biopsy, rendering many of the old debates irrelevant and rapidly quickening the pace of uptake of SN biopsy. In this instance, the improvements in survival for patients treated with adjuvant systemic therapy and the listing of these therapies on the government-funded Pharmaceutical Benefits Scheme helped to accelerate change. Importantly, medical oncologists acting as expert opinion leaders have played an important role in disrupting the social and knowledge boundaries between the professional groups and communities of practice in melanoma management by highlighting the potential benefits of adjuvant systemic therapy to patients with Stage III melanoma. An expert opinion leader in healthcare has been defined as a credible authority (often an academic or senior specialist) able to explain the evidence and respond convincingly to challenges and debate, or alternatively as a person whose support for an initiative or innovation is itself sufficient endorsement [20]. As Rogers’ classic theory of diffusion highlights, the potential adopters’ perceptions of opinion leaders’ reactions to an innovation is a key contributor to the innovation’s diffusion or lack thereof [36, 45]. Although Roger’s other factors, the pros and cons of the innovation and how proponents and opponents frame the meaning of the innovation, have also been crucial to the adoption of the SN biopsy guidelines, it would appear that it was the potential adopter’s perceptions of key opinion leaders and their reactions to SN biopsy that have been most influential.

Although the communities of practice to which dermatologists, surgeons and primary care physicians belonged appeared to be responsible for slowing the spread of knowledge, they can also be viewed in a more constructive light. It was suggested by some of the key informants that the push to adopt SN biopsy took place in advance of sufficient evidence supporting its use, resulting in reservations and at times challenges to the veracity of the SN biopsy guideline recommendations, particularly from some dermatologists. It is possible that the differences in opinion that existed within these different communities of practice may have helped to drive the generation of new research studies that have ultimately helped to clarify what is appropriate use of SN biopsy and our understanding of which melanoma patients will derive most benefit from it.

This study highlighted how it can be difficult to gauge whether an innovation such as SN biopsy has been successfully and appropriately adopted. Adoption is a multi-factorial process, not a one-off event, and may take time. As evidence accumulates and the field of medicine itself advances, the role and function of an innovation can change, impacting on what is deemed appropriate adoption. As our findings indicate, SN biopsy is an example of an intervention that has cycled through different phases of implementation, de-implementation and re-implementation [26, 46] as the evidence to support its use has matured and as new treatments for melanoma management have emerged, shifting practitioners’ perceptions of what is appropriate use of SN biopsy.

Finally, our study highlighted that the provenance of guidelines can be problematic, with some indicating that the reluctance to accept and adopt the guidelines may have been in part because of the perception that they had been developed primarily by one group of clinicians (surgeons) yet applied to the clinical practice of several other groups of clinicians (dermatologists and primary care physicians). Others have reported that guidelines developed by specialists may seem self-serving, biassed, and threatening to other specialists or generalists [47]. Fairhurst and Guby have reported that GPs often use local guidelines produced by local specialists known to them rather than using national guidelines, even when these national guidelines were written by acknowledged experts, reinforcing what has been reported about the importance of collectively reinforced, internalised, tacit ‘mindlines’ as opposed to guidelines [20, 31, 48].

Given that the SN biopsy guideline recommendations apply to clinicians from different professional groups (surgery, dermatology, primary care) and that adherence has the potential to shift work across professional boundaries altering a clinician’s workflow, patient care role and revenue, it is perhaps not surprising that acceptance, adoption, and adherence to the SN biopsy guideline recommendations has been challenging.

### Strengths and limitations

Various strategies were used throughout the analysis process to enhance the rigour and trustworthiness of the findings. Regular meetings were held to discuss the interpretation of codes and themes, sharing of memos and notes, co-coding of qualitative data, data triangulation (using multiple data collection methods and sources including interviews with a range of key informants, news stories, and field notes) as well as to consider disparate views ensuring balanced investigation of service provider perspectives. Provision of ample and rich quotes from participants enhanced the connection between data and conclusions. However, several of the respondents were selected on the basis of their public pronouncements on SN biopsy. The views of these people and those they nominated represent strong views and are not necessarily representative of all dermatologists, surgeons, primary care physicians or other clinicians involved in melanoma management.

## Conclusions

Clinical practice is determined as much by what a clinician knows as by the norms within their community of practice, and by the meaning that an innovation holds for clinicians. This meaning differs depending on professional group affiliation or community of practice and evolves with time, changing with the emergence of new evidence and new opinion leaders. Adoption of SN biopsy in Australia over the past 20 years has involved a process of assessing continually evolving evidence from clinical trials followed by review and reframing by clinicians from different specialty areas involved in the management of primary melanoma of the evidence for SN biopsy. It is anticipated that we will continue to see change in the uptake of SN biopsy as knowledge of the role of adjuvant therapies in melanoma management diffuses, or is actively disseminated, amongst these communities of practice by these emerging opinion leaders. Any intervention aimed at improving uptake or adherence to SN biopsy recommendations would benefit from a co-design approach to ensure appropriate engagement of key existing and emerging opinion leaders who are familiar with the evidence on which those recommendations are based.

## Supplementary Information

**Additional file 1:** Interview guide. **Table S1.** Guideline factors identified in stakeholder data as impacting on use of sentinel node biopsy for patients with melanoma. **Table S2.** Health professional factors identified in stakeholder data as impacting on use of sentinel node biopsy for patients with melanoma. **Table S3.** Patient factors identified in stakeholder data as impacting on use of sentinel node biopsy for patients with melanoma. **Table S4.** Professional interaction factors identified in stakeholder data as impacting on use of sentinel node biopsy for patients with melanoma. **Table S5.** Incentives and resource factors identified in stakeholder data as impacting on use of sentinel node biopsy for patients with melanoma. **Table S6.** Social factors identified in stakeholder data as impacting on use of sentinel node biopsy for patients with melanoma.

## Data Availability

The de-identified datasets analysed during the current study are not publicly available due to conditions of ethics approval but may be available from the corresponding author on reasonable request subject to appropriate approval from an ethics committee.

## Abbreviations

AJCC: American Joint Committee on Cancer
PBS: Pharmaceutical Benefits Scheme
SN: Sentinel node
