# Supplementary material for: Factors influencing acceptance, adoption and adherence to sentinel node biopsy recommendations in the Australian Melanoma Management Guidelines: a qualitative study using an implementation science framework
Source: Implement Sci Commun. 2022 Oct 1;3:103. doi: 10.1186/s43058-022-00351-w (PMC9526940; doi:10.1186/s43058-022-00351-w)
Supplement: Supplementary file 1 — Additional file 1: Interview guide. Table S1. Guideline factors identified in stakeholder data as impacting on use of sentinel node biopsy for patients with melanoma. Table S2. Health professional factors identified in stakeholder data as impacting on use of sentinel node biopsy for patients with melanoma. Table S3. Patient factors identified in stakeholder data as impacting on use of sentinel node biopsy for patients with melanoma. Table S4. Professional interaction factors identified in stakeholder data as impacting on use of sentinel node biopsy for patients with melanoma. Table S5. Incentives and resource factors identified in stakeholder data as impacting on use of sentinel node biopsy for patients with melanoma. Table S6. Social factors identified in stakeholder data as impacting on use of sentinel node biopsy for patients with melanoma. [file 43058_2022_351_MOESM1_ESM.docx]

# SUPPLEMENTARY FILE

Interview guide

**Melanoma management study: Interview guide for experts and stakeholders**

The interview guide does not need to be followed precisely. The aim is to tailor questions to the participant’s experiences and involvement in SLN biopsy, to their responses to questions asked earlier in the interview, and to any aspects that appear particularly important to the participant.

**[If the interview is via telephone:]**

Hello, can I please speak with [participant’s name].

Thank you for taking the time to talk with me today. My name is [researcher’s name], and I’m part of the melanoma research team with the University of Sydney. We scheduled this time for an interview to discuss your thoughts about managing the care of melanoma patients. I expect that the interview will last around 20-30 minutes. Is now still a good time to talk?

[*If yes, then continue; If no, then reschedule*]

I would like to make an audio recording, just to make sure that I can accurately record what we talk about. Is this ok? Everything that is said today will be kept strictly confidential.

*[If the participant agrees, start recording]*

Do you have any questions about this interview before we begin?

1. Can you tell me about your involvement / your organisation’s involvement in melanoma management?
   - Can you tell me about your involvement / your organisation’s involvement in SLN biopsy for melanoma?
   - Can you tell me about how you / your organisation regards SLN biopsy for melanoma?
2. Think back to the 1990s when SLN biopsy was first introduced.
   - Did you have any particular views on SLNB?
   - How have they changed?
3. What are some of the issues relating to SLN biopsy?
   - What are the difficulties in implementation of SLN biopsy?
4. Views on current SLN biopsy guidelines / recommended practice.
   - I know you have written about SLN biopsy ..... can you expand on that? [NB if participant seems to be reiterating the ‘party’ line of the organisation they work for, then remind them the interview is confidential and say ‘you don’t have to answer this question, but I wonder how your personal views compare with the organisation’s policies and practices, and whether there might be any points of difference?]
5. There are many different ideas about SLN biopsy, can you comment on these?
   - There are some who hold quite extreme views on SLN biopsy. How do you respond to these views?
   - What do you think drives these views?
   - Can you see any good in their position? Is there anything about what you think such people are trying to achieve that might lead to some common ground with yourself / your organisation?
6. What would it take to make changes happen in relation to use of SLN biopsy?
   - What might be the barriers to change?
   - What conditions do you think would need to be met before SLN biopsy became widely implemented?
   - What conditions do you think would need to be met before SLN biopsy became discontinued?
   - What do you think will happen in relation to use of SLN biopsy in the next 5 years / 10 years?
7. What process would you like to see Australia follow in relation to SLN biopsy? [NB: Feed in comments / suggestions that other interviewees have made, e.g. researchers, consumers, clinicians; alternatives to SLN biopsy; guidelines or recommendations in other countries such as UK, US].

Table S1 Guideline factors identified in stakeholder data as impacting on use of sentinel node biopsy for patients with melanoma

| **Flottorp determinant** | **Explanation** | **Key points identified in analysis** | **Illustrative quotes** |
| --- | --- | --- | --- |
| Quality of evidence supporting the intervention | The quality of the evidence supporting the recommendation and whether it has been assessed appropriately | Distrust of the validity/strength of the evidence base  Beliefs among some clinicians that evidence base for SN biopsy was unclear and open to interpretation | *‘**A little bit of questionable statistics and the pushing that this is - and the survival benefit, I think that's where [the opposing views on SN biopsy] come from. Dermos felt compelled to argue.’ ID04 Dermatologist*  *‘The context that's led to so much tension and controversy about [SN biopsy] is that just like elective lymph node dissection, the surgical literature is just full of spin and promotion that's not justified by the evidence. It's not balanced and that generates a negative response on the other side.’ ID05 Dermatologist*  *‘The final analysis of MSLT-I tried to engineer a survival benefit from the data in ways that were not generally accepted - retrospective latent subgroup analyses that were in any trial not really accepted - and then used a statistical analysis that they've generated themselves, wasn't validated or accepted or understood.’ ID05 Dermatologist*  *‘A number of people have commented that the selective reporting [of results from MLST-I] and the post-hoc analysis of the data has severely diminished the quality of that trial.’ ID19 Pathologist*  *‘Now that we have the results of [the adjuvant systemic therapy] trials then we recommend [SN biopsy] more strongly.’ ID6 Dermatologist*  *‘A lot of the people in the primary care sector and a lot of people doing a lot of skin work felt that actually [the results of MLST-I] didn't justify [recommending SN biopsy]. Because of no survival outcomes, it wasn't clearly demonstrated that there was good survival outcome, that again was it something that should be routinely offered to patients.’ ID12 Senior executive (surgical and GP background)* |
| Source of recommendation | The credibility of the organisation(s) and people who made the recommendation with the targeted healthcare professionals | Suspicious of guidelines not from own disciplinary group  Perceived conflicts of interest among those involved in the guideline development process | *‘I think just the perception from outside of [the guidelines being chaired by a surgeon] is probably not favourable. So I think probably it would have been ideal to have declared that as a conflict and probably stepped aside from any discussions around sentinel node biopsy.’ ID6 Dermatologist* |
| Compatibility of recommendation to current practice | The extent to which the recommended behaviour fits with current practices and whether it might disrupt current workflow | Some disagreement over the recommendation to refer to a surgeon for discussion of SN biopsy, especially among dermatologists | *‘One of the things actually in the guidelines that people have taken exception to, particularly some dermatologists, is that the procedure itself should be discussed with the person who does the procedure. Or the patient should be offered the opportunity to do that which I think is essential, actually. I think it's just good practice to offer that referral.’ ID6 Dermatologist*  *‘It's not one of the recommendations but as a practice point [referral to* *surgeon] gets under the skin of dermatologists. [Dermatologists are concerned as] it says we have to send every patient with melanoma this thick to a surgeon or we may be negligent.’ ID15 Dermatologist* |

Table S2 Health professional factors identified in stakeholder data as impacting on use of sentinel node biopsy for patients with melanoma

| **Flottorp determinant** | **Definition** | **Key points identified in data analysis** | **Illustrative quotes** |
| --- | --- | --- | --- |
| Awareness and familiarity with recommendations | The extent to which the targeted healthcare professionals are aware of and familiar with the recommendation | Difficulty in making guidelines known to the diverse range of specialists (surgeons, dermatologists, GPs) working across different settings (primary, secondary and tertiary care) involved in melanoma management | *’I’ve interacted with literally thousands of GPs. Most of them don’t know what they’re doing in [relation to SN biopsy recommendations]. They don’t see enough cases to know what they’re doing … More and more skin cancer doctors do know what they’re doing, but there’s a lot of them that also don’t know what they’re doing.’ ID11 GP.* |
| Agreement with the recommendation | The extent to which the targeted healthcare professionals agree with the recommendation | Although stakeholders on the whole were in agreement with SN recommendation, it was acknowledged that target healthcare professionals did not necessarily all agree with recommendation | *‘I don't like the phrase extremism, what you have is just, I think, two groups of people who have looked at the same literature and reached different conclusions. Then you have a large group of people in the middle who are happy to be guided by the experts.’ ID03 Dermatologist*  *‘There were some serious arguments, particularly in the dermatological world against sentinel node.’ ID17 Dermatologist*  *‘If you have a dermatologist who's antagonistic to [SN biopsy] and they discuss it, the patient will have a very different discussion than being able to talk to a surgeon.’ ID22 Surgeon* |
| Expected outcomes | The extent to which the targeted healthcare professionals believe that adherence with the recommendation will lead to desired outcomes | Prior to the introduction of systemic adjuvant therapies, some clinicians believed risks of SN biopsy (avoidable morbidity, costs, lack of survival benefit) outweighed possible benefits (prognostic information). Survival benefits associated with systemic therapies meant many clinicians now viewed SN biopsy more favourably | *‘We encourage [SN biopsy] more so these days now that it leads to less surgery and maybe more medical therapy’. ID4 Dermatologist*  *‘A lot of the people in the primary care sector and a lot of people doing a lot of skin work felt that actually [the prognostic information] didn't justify [doing a SN biopsy]. Because of no survival outcomes, it wasn't clearly demonstrated that there was good survival outcome, that [it was] something that should be routinely offered to patients.’ ID11 GP* |

Table S3 Patient factors identified in stakeholder data as impacting on use of sentinel node biopsy for patients with melanoma

| **Flottorp determinant** | **Definition** | **Key points identified in data analysis** | **Illustrative quotes** |
| --- | --- | --- | --- |
| Patient preferences | Patients’ values in relationship to professional values or those in the recommendation | Patient preferences may differ from those of the clinician, especially if the clinician has strong views on role of SN biopsy | *‘If you have a dermatologist who's antagonistic to [SN biopsy] and they discuss it, the patient will have a very different discussion than being able to talk to a surgeon.’ ID22 Surgeon*  *‘Patients want to know their correct stage. That's the bottom line. You want to know what stage you are and do I need any further treatment: drugs, radiation, surgery.’ ID26 Melanoma consumer representative* |

Table S4 Professional interaction factors identified in stakeholder data as impacting on use of sentinel node biopsy for patients with melanoma

| **Flottorp determinant** | **Definition** | **Key points identified in data analysis** | **Illustrative quotes** |
| --- | --- | --- | --- |
| Communication and influence | The extent to which the targeted healthcare professionals’ adherence is influenced by professional organisations, professional networks, prevailing norms (opinions of colleagues) or opinion leaders (or champions or other influential people) | Role of communities of practice  Role of MDTs and centres of expertise in melanoma on influencing beliefs and practice  Role of perceived norms held by different specialties (e.g. dermatologists versus surgeons)  Role of senior colleagues and specialists in guiding opinions of clinicians | *‘I think unfortunately the overstating the findings from the MSLT-I trial has done proponents of sentinel node biopsy a disservice and has bred this mistrust of the procedure itself and anyone who advocates for the procedure. So I feel that there is this cynicism of anything that then is put forward by the pro-sentinel node group which is unhelpful and it has become quite personal to a lot of people and has become quite emotive.’ ID6 Dermatologist*  *‘Surgeons have for a very long time … driven sentinel node biopsy quite strongly, and perhaps historically overstated the advantages of it. I think that there was a degree of scepticism which many people then found when the research came out that it wasn't necessarily giving that big survival advantage.’ ID12 Surgeon*  *‘I think a lot of people who have been involved in this trenchant opposition just don't have that opportunity or have turned their back on that opportunity of being part of a multi-disciplinary team.’ ID15 Dermatologist*  *‘[Differences in opinions about SN biopsy arise] because that's the way their colleagues have spoken and they tend to follow their colleagues, [for example] surgeons listen to surgeons.’ ID14, GP* |

Table S5 Incentives and resource factors identified in stakeholder data as impacting on use of sentinel node biopsy for patients with melanoma

| **Flottorp determinant** | **Definition** | **Key points identified in data analysis** | **Illustrative quotes** |
| --- | --- | --- | --- |
| Financial incentives and disincentives | The extent to which patients, individual health professionals and organisations have financial incentives or disincentives to adhere | Vested interests  Turf protection  Reluctance of GPs and dermatologists to refer patients (e.g. to surgeon) in case this means losing the patient (and the procedure)  Perception among some that surgeons are promoting a procedure that they stand to gain from financially | *‘I think the conclusions [about utility of SN biopsy] and the driving force out of the US has been probably driven by money because the data did not show overall improvement in survival.’ ID10 Dermatologist*  *‘I think it is the view amongst some that they're being dealt out of melanoma management. So dermatologists do feel threatened.’ ID15 Dermatologist*  *‘There are vested interests. It is possible that [dermatologists and GPs] don't wish to refer patients to the surgeon ... You have to do the wide excision at the same time. So, in other words, they're losing business, losing patients if they refer them.’ ID22 Surgeon*  *‘Subset analyses are just not done and that annoyed people. It’s very annoying because then if you’re saying that there’s a survival benefit, you’ve got to send them. So that controversy was annoying.* *Those claims were really annoying and most dermatologists in the world thought that that was – and I’ll say public health academics – really thought those claims were made by people who wanted to perform the procedure. That there was either a* ***financial reason*** *or they had a bias of doing it.’ ID17 Dermatologist* |
| Non-financial incentives and disincentives | The extent to which patients, individual health professionals and organisations have non-financial incentives or disincentives to adhere, e.g. personal recognition or appreciation (from managers, colleagues or the community), career development (e.g. opportunities to specialise or be promoted) | Professional identity  Professional standing  Personal recognition  Academic reputation | *‘The perception that I'm essentially looking after the patient, I've been doing the skin checks and the surveillance and all the rest of it. Actually, I can do the wide local excision, so I can manage this patient completely. The only thing which throws a spanner in the works is the sentinel node biopsy.’ ID12 GP*  *‘Sometimes I have the impression it's really surgeon against non-surgeon and I don't think that this is good and some of them saying they do this because of money. I don't think that this is the case. That's a ridiculous argument. I think it's more that when you do something well you see it positive and human nature that others don't do it and they have a different view and the topic is simply too complex.’ ID2 Dermatologist*  *‘I think a big part of this is that doctors are very afraid to stop doing something they've been doing for a long time. I think this is a big part of it.’ ID1 Dermatologist*  *‘The surgeons that have taken years to learn the technique and have been involved in lots of studies that seem to want to encourage it no matter what. Then the more pragmatic physicians who are trying to understand the place of the biopsy and you get different views on different continents.’ ID5 Dermatologist*  *‘‘Financial interests was at the bottom of [eminent surgeon’s] list [of reasons why surgeons continue to promote SN biopsy], but top of the list was academic reputation. They don't want to be proven wrong … There's ego, there's academic reputation, they don't want to be proven wrong. The other reasons [eminent surgeon] gave was inertia, like we've always done it this way, we're just going to keep on doing what you're always doing, which you've done for the last 20, 30 years. You don't want to change your methods or approach. Other reasons were loss of face and things like that. There's a lot of academic reputation involved.’ ID11 GP* |
| Assistance for clinicians | The extent to which clinicians have the assistance they need to adhere, such as checklists, patient information, decision aids, decision support, or clinical supervision | SN recommendations (and melanoma guidelines in general) are relatively inaccessible to GPs – there is a need for a simple-to-understand summary / decision making tool / algorithm | *‘A lot of [GPs] … make it up as they go along and a good part of that is because the guidelines are very difficult to access, and read, and understand and appreciate.’ ID11 GP* |

Table S6 Social factors identified in stakeholder data as impacting on use of sentinel node biopsy for patients with melanoma

| **Flottorp determinant** | **Definition** | **Key points identified in data analysis** | **Illustrative quotes** |
| --- | --- | --- | --- |
| Payer or funder policies | The extent to which payer of funder policies may affect implementation of necessary changes | Fee for service: procedures generate more income than consultation | *‘In the Australian system you get paid for a wide excision, yeah, but you don't get paid for half an hour or one-hour complex consultation process.’ ID2 Dermatologist* |
| Influential people | The extent to which influential people facilitate or hinder implementation of necessary changes | Role of not just research data but commentaries, editorials, presentations in positively or negatively influencing beliefs about SN biopsy and credibility of recommendations / guidelines | *‘The dermatology community, who have resisted [SN biopsy] without actually providing any data, have claimed that it's not of value and have actively campaigned to encourage patients not to have it.’ ID22 Surgeon*  *‘If the proponents hadn't been so strong in the first instance and claiming too much for [SN biopsy], then they may not have got such a strong reaction on the other side ... You get this sort of polarised situation where there's the zealot unreasonably promoting [SN biopsy] and the sceptics that are unreasonably rejecting [SN biopsy] and the truth is somewhere in the middle.’ ID05 Dermatologist* |
